# Acceptability and perceived harm of calorie labeling and other obesity policies: A cross‐sectional survey study of UK adults with eating disorders and other mental health conditions

**DOI:** 10.1002/eat.24031

**Published:** 2023-07-28

**Authors:** I Gusti Ngurah Edi Putra, Megan Polden, Lettie Wareing, Eric Robinson

**Affiliations:** ^1^ Department of Psychology, Institute of Population Health University of Liverpool Liverpool UK; ^2^ Department of Primary Care and Mental Health, Institute of Population Health University of Liverpool Liverpool UK; ^3^ NIHR Applied Research Collaboration North West Coast Liverpool UK; ^4^ Department of Psychology Lancaster University Lancaster UK

**Keywords:** calorie information, eating disorders, mental health, obesity policy, public health policy

## Abstract

**Objective:**

We assessed perceptions of recently proposed UK obesity policies (mandatory calorie labeling, banning of advertisements of unhealthy food and drinks online and before 9 pm on TV, and banning “buy one get one free” deals for unhealthy food and drinks) in people with an eating disorder (ED) and other mental health conditions.

**Method:**

A total of 1273 participants with a self‐reported lifetime mental health condition (*N* = 583 with an ED) completed an online survey in September–November 2022. Multinomial logistic regression was used to examine support for and potential adverse effects of policies in participants with and without an ED. A qualitative analysis of the potential effects of the policy on current ED symptoms was also conducted to better understand how and why policies may be damaging or beneficial.

**Results:**

Participants with an ED had a lower level of support for the implementation of the calorie labeling policy compared to those without an ED (43% vs. 58%). Half of the participants with an ED (55%) reported that labeling may worsen their ED symptoms. Qualitative data indicated perceived potential harm (e.g., a gateway to relapse, negative effects on mood) and perceived benefits (e.g., feeling informed and reassured) of calorie labeling in participants with an ED. No differences in support or perceived harms of the other two policies were observed between participants with versus without an ED.

**Discussion:**

Future studies are warranted to explore the potential effects of calorie labeling and how to mitigate negative impacts on people with an ED.

**Public Significance:**

This research is the first to assess the perceptions of UK obesity‐related policies in people with an ED and other mental health conditions. Participants with an ED (vs. without) were more likely to disagree with the government implementing the calorie labeling policy. These findings highlight the potentially harmful effects of calorie labeling in people with an ED and the need for future research to understand how to mitigate negative impacts.

## 1 INTRODUCTION

Obesity, a condition of excess fat accumulation indicated by having a body mass index (BMI) ≥ 30 kg/m^2^ (WHO, 2021), is considered a major public health problem in the United Kingdom and worldwide. The prevalence of obesity nearly doubled from 15% to 28% in England from 1993 to 2019 (Baker, 2022). In addition, 23% of children aged 10–11 years in England had obesity in 2021/2022 (NHS Digital, 2022). Living with obesity is associated with adverse psychological, musculoskeletal, cardiovascular, and cancer outcomes (Reilly et al., 2003) and accounts for around 10% of years of life lost in England (Steel et al., 2018). Obesity‐related health issues are estimated to cost the NHS more than £6 billion per year (Scarborough et al., 2011).

The UK Government has recently proposed new public health policies designed to tackle obesity (Department of Health and Social Care, 2018a, 2020b). These policies included a proposal to mandate calorie labeling in large‐scale out‐of‐home food outlets (OHFO). The policy applies to all large businesses with ≥250 employees in England where food or drink is prepared for immediate consumption (cafes, restaurants, takeaways) and was implemented in 2022. Specifically, calorie labeling in kilocalories (with reference to the recommended daily intake of adult women of 2000 kcal) is required across all points of choice and order points in food outlets (Department of Health and Social Care, 2018b, 2020a). Further recently proposed policies include the banning of advertisements of unhealthy food and drink both online, and before 9 pm on TV and banning “buy one get one free” deals for unhealthy food and drinks (Department of Health and Social Care, 2020b; Iacobucci, 2022).

Calorie labeling policy will provide consumers with easy access to calorie information, which may impact consumer purchasing behavior leading to wider impacts on health and obesity levels (Department of Health and Social Care, 2020a). However, the introduction of mandatory calorie labeling in UK OHFO has some public concern (Polden et al., 2023) and could be damaging to people with mental health conditions, particularly people with an eating disorder (ED) (Kaur et al., 2022; Olsen, 2021). Specifically, Beat, the UK ED charity opposed the implementation of mandatory calorie labeling after warning that calorie labeling on menus “risks exacerbating all eating disorders” (Beat Eating Disorders, 2020). Furthermore, it has been argued that calorie labeling will be harmful to people with a historic or current ED by increasing anxiety and feelings of guilt over calories, potentially leading to worsening ED symptoms (Beat Eating Disorders, 2020).

Over the past few years, the prevalence of ED in the United Kingdom has risen substantially. Based on a report by a coalition working to tackle ED, The Hearts Minds Genes Coalition for Eating Disorders (2021), it was estimated that 1.5–2.4 million people may have an ED and this may cost the United Kingdom between £7.5 and £11.2 billion in 2020. Moreover, hospital admissions for people with an ED have increased by 84% over the last 5 years according to a report by the Royal College of Psychiatrists (2022b). Consequently, given the prevalence and risks associated with ED, understanding whether current and future obesity policies may be harmful to people with an ED is of importance.

There is limited evidence examining the direct effect of calorie labeling on people with an ED. A study by Haynos and Roberto (2017) recruiting undergraduate female students in the United States found that when making hypothetical food choices with calorie labeling presented the menu, participants who met questionnaire cut‐offs for anorexia nervosa and bulimia nervosa symptomatology ordered meals with fewer calories, but participants with a tendency towards binge‐ED selected meals with more calories. The presence of calorie labeling may have resulted in food choices associated with maintaining the current ED symptomatology, though other studies have not reached the same conclusion (Lillico et al., 2015; Robinson et al., 2022). Furthermore, even though there is comorbidity between obesity and ED (e.g., binge‐ED) (Darby et al., 2009) and other mental health conditions (e.g., depression) (Khanolkar & Patalay, 2021), there is very limited evidence on perceptions of public health policies to address obesity in people with an ED and other mental health conditions. Given that national obesity policies like menu calorie labeling have recently been introduced in the US and England and other countries are considering adopting similar policies (Obesity Action Scotland, 2022; Royal College of Paediatrics and Child Health, 2022), there is an urgent need to understand potential unintended consequences for people living with mental health conditions, including EDs.

The primary aim of the present research was to investigate acceptability and perceived harm of mandatory calorie labeling on menus in UK adults with an ED and mental health conditions. For comparative purposes, we also examined other recent UK obesity‐related public health policies (i.e., banning advertisements of unhealthy food and drinks online and before 9 pm on TV; banning “buy one get one free” deals for unhealthy food and drinks). In addition to participants with a history of ED, we also sampled participants with a current or past history of other mental health conditions as research to date on obesity policy has neglected the views of people with an ED and other mental health conditions (Beeken & Wardle, 2013; Fatemi et al., 2021; Petrescu et al., 2016).

## 2 METHOD

### 2.1 Participants

Participants were recruited online from Prolific Academic and social media (e.g., Facebook, Twitter) (September–November 2022). Prolific Academic is a widely used participant recruitment site for online research (https://www.prolific.co/). We calculated a minimum sample size of 865 using G*POWER 3.1.9.7 (Demidenko, 2007; Faul et al., 2009) (see Table S1). Participants were eligible if they were aged ≥18 years, lived in the United Kingdom, were fluent in English, and had ever been diagnosed with a mental health condition(s) including an ED by a medical practitioner. We recruited 1273 participants from Prolific Academic (*n* = 1199) and social media (*n* = 74), of which, 583 participants had an ED diagnosis. The ethics approval was from the Central University Research Ethics Committee of the University of Liverpool (Reference: 11397) and informed consent was obtained from all participants.

### 2.2 Measures

We collected information on socio‐demographic characteristics: age (years), gender (male, female, other), and ethnicity (White, non‐White). Educational level was recoded into degree‐level education (e.g., degree, foundation degree, HND or HNC level 4 and above, teaching or nursing) and below degree‐level education following the UK 2021 Census (Office for National Statistics, 2020). Equivalised household income was calculated by adjusting annual household disposable income with the household size and composition (Office for National Statistics, 2015), and then transformed into tertiles. BMI in kg/m^2^ was used to define participants into underweight (< 18.5), normal weight (18.5–24.9), overweight (25–29.9), and obesity (≥ 30) categories. Self‐reported and objectively measured BMI is highly correlated in the general population (Wright et al., 2015), though people with disordered eating tend to overestimate their body weight (Conley & Boardman, 2007; Hagman et al., 2015).

Participants completed a 7‐item Eating Disorder Examination Questionnaire (EDE‐Q7) that consists of three subscales: dietary restraint (three items), shape/weight overvaluation (two items), and body dissatisfaction (two items) (Jenkins & Davey, 2020). A summary score was constructed by averaging responses on a 7‐Likert point scale from 0 to 6, with higher scores indicating greater symptomatology. Findings from exploratory factor analysis indicated that EDE‐Q7 items did not all load onto a single factor (see Table S2). We therefore calculated an average score of four items from the shape/weight overvaluation and body dissatisfaction subscales to indicate overall ED symptomatology as these items loaded on the same factor. This four‐item measure had a good internal consistency (see Table S2). A four‐item Patient Health Questionnaire (PHQ‐4) (Kroenke et al., 2009; Stanhope, 2016) was administered to assess general mental health. A sum score ranges from 0 to 12 with a higher score indicating greater anxiety and depressive symptoms. PHQ‐4 had good construct validity and internal consistency reliability (see Table S3). Participants also reported whether they had been diagnosed with any ED and/or mental health conditions by a medical practitioner. Participants who responded “yes” were asked to select which ED (e.g., anorexia nervosa) and/or mental health conditions (e.g., generalized anxiety disorder) they had been diagnosed with and the status of their current symptoms (e.g., “I had an eating disorder(s) in the past and have no current symptoms”).

Five‐point Likert scale questions with rating scales from strongly disagree to strongly agree were included to assess participants' perceptions of obesity policies. Participants also rated 6‐point Likert scale questions from much worse to much better on the potential effects of these policies on their current mental health and/or ED symptoms. As our focus here is on calorie labeling, items that were used to examine opinions on calorie labeling are presented in the main document, and items to measure opinions on the other two policies are available in supplementary materials. Free text response questions allowed participants to express their opinions on why these policies might be damaging to their symptoms.

### 2.3 Data analysis

We used descriptive statistics to compare the levels (%) of acceptability/support and perceptions of the policies between participants with and without an ED. Multinomial logistic regression was used to examine whether participants with an ED (vs. without) were more likely to disagree with calorie labeling and perceive its negative effects, controlling for sociodemographic covariates and BMI category. If we found statistically significant associations, we further adjusted the associations for current ED and mental health symptomatology. To conduct multinomial logistic regressions, we recoded participants' responses into three categories: disagree (for “strongly disagree” and “disagree,”) neutral (for “neither agree nor disagree”), and agree (for “agree” and “strongly agree”). Likewise, the responses to the perception of the effects of policy on current ED or mental health symptoms were grouped as worse (for “somewhat worse” and “much worse”), neutral (for “no different” and “unsure”), and better (for “somewhat better” and “much better”). We examined the likelihood of being disagree or agreeing, relative to neutral, or perceiving that the policy would make the current symptoms worse or better, relative to neutral. Findings from multinomial logistic regression were presented as relative risk ratio (RRR) along with 95% confidence intervals (CI) and *p*‐value.

If differences in perceptions of the calorie labeling policy by ED diagnosis were evident, follow‐up analyses were conducted in participants with an ED only. Using multinomial logistic regression, we examined whether the views on calorie labeling differ by (1) ED symptoms (current ED diagnosis and experiencing symptoms versus past ED and no/some lingering symptoms), and (2) specific ED (anorexia nervosa, bulimia nervosa, and binge‐ED) and mental health conditions (generalized anxiety disorder, major depressive disorder) predominantly reported by participants (see Table 1), controlling for the same covariates as the full‐sample analyses. Because of comorbidities, separate binary variables (yes; no) were developed for EDs and mental health conditions.

**TABLE 1:** Characteristics of the participants.

Variables	Participants with an ED diagnosis (*n* = 583)		Participants without an ED diagnosis (*n* = 690)		Total (*n* = 1273)	
	Mean (SD)	*n* (%)	Mean (SD)	*n* (%)	Mean (SD)	*n* (%)
*Socio‐demographic characteristics*						
Age	31.45 (9.49)	581	37.26 (11.76)	684	34.59 (11.15)	1265
Gender		581		687		1268
Man		120 (20.65)		229 (33.33)		349 (27.52)
Woman		438 (75.39)		445 (64.77)		883 (69.64)
Non‐binary and other		23 (3.96)		13 (1.89)		36 (2.84)
Ethnicity		580		686		1266
Non‐White		59 (10.17)		55 (8.02)		114 (9.00)
White		521 (89.83)		631 (91.98)		1152 (91.00)
Education		580		686		1266
Below than degree level		206 (35.52)		286 (41.69)		492 (38.86)
Degree level		374 (64.48)		400 (58.31)		774 (61.14)
Equivalised household income (in £)	25,538 (24,811)	446	25,046 (23,594)	585	25,259 (24,118)	1031
Tertile 1 (lowest)		146 (32.74)		198 (33.85)		344 (33.37)
Tertile 2 (middle)		152 (34.08)		211 (36.07)		363 (35.21)
Tertile 3 (highest)		148 (33.18)		176 (30.09)		324 (31.43)
BMI	26.57 (8.71)	487	28.12 (7.31)	603	27.43 (8.00)	1090
Underweight (< 18.5)		71 (14.58)		25 (4.15)		96 (8.81)
Normal weight (18.5–24.9)		183 (37.58)		208 (34.49)		391 (35.87)
Overweight (25–29.9)		100 (20.53)		181 (30.02)		281 (25.78)
Obesity (≥ 30)		133 (27.31)		189 (31.34)		322 (29.54)
*Mental health symptoms and conditions*						
ED symptoms^a^	4.81 (1.31)	574	4.09 (1.73)	685	4.42 (1.60)	1259
PHQ‐4 for anxiety and depressive symptoms	7.91 (3.33)	578	6.54 (3.61)	689	7.16 (3.55)	1267
Diagnosed ED conditions^b^		583				
Anorexia nervosa		187 (32.08)				
Avoidant/restrictive food intake disorder		51 (8.75)				
Binge‐eating disorder		196 (33.62)				
Bulimia nervosa		144 (24.70)				
OSFED or UFED		129 (22.13)				
Status of ED symptoms		571				
Past ED and no current symptoms		66 (11.56)				
Past ED and some lingering symptoms		313 (54.82)				
Currently ED and experience symptoms		192 (33.63)				
Diagnosed other mental health conditions^b^		523		690		1213
Generalized anxiety disorder		405 (77.44)		518 (75.07)		923 (76.09)
Bipolar disorder		20 (3.82)		20 (2.90)		40 (3.30)
Body dysmorphic disorder		36 (6.88)		11 (1.59)		47 (3.87)
Borderline personality disorder		57 (10.90)		26 (3.77)		83 (6.84)
Major depressive disorder		404 (77.25)		555 (80.43)		959 (79.06)
Obsessive‐compulsive disorder		71 (13.58)		66 (9.57)		137 (11.29)
Panic disorder		52 (9.94)		57 (8.26)		109 (8.99)
Post‐traumatic stress disorder		94 (17.97)		73 (10.58)		167 (13.77)
Schizophrenia		1 (0.19)		3 (0.43)		4 (0.33)
Other		43 (8.22)		32 (4.64)		75 (6.18)
Status of other mental health (MH) symptoms		522		689		1211
Past MH with no current symptoms		14 (2.68)		49 (7.11)		63 (5.20)
Past MH and some lingering symptoms		135 (25.86)		247 (35.85)		382 (31.54)
Current MH and experience symptoms		373 (71.46)		393 (57.04)		766 (63.25)

Abbreviations: BMI, body mass index; *n*, number of participants; OSFED, other specified feeding or eating disorder; PHQ‐4, 4‐item Patient Health Questionnaire (the total score ranges from 0 to 12); SD, standard deviation; UFED, unspecified feeding or eating disorder; %, percentage.

^a^ Average score (ranging from 0 to 6) of four items (shape/weight overvaluation and body dissatisfaction subscales) that loaded on the same factor (see supplementary materials).

^b^ Some participants reported having more than one condition; 523 participants with ED also experienced a mental health condition(s).

All the analyses above were replicated on public health policies on restricted marketing and price promotions. If differences in views on these policies were evident by ED diagnosis, separate analyses were carried out for people with an ED only. For main regression analyses of the full sample with adjustments for sociodemographic characteristics and BMI category, we set *p* < .05. Meanwhile, for secondary analyses of full‐sample analyses with additional adjustments for the current ED and mental health symptomatology and for sub‐group analyses of participants with an ED only, we adjusted *p*‐values to a lower threshold to avoid the risk of drawing a false‐positive conclusion (Type I error) when testing multiple hypotheses. Using the Bonferroni correction (Lee & Lee, 2018; Sedgwick, 2012) with more than 50 secondary tests including the analysis to address missing observations, *p* < .001 (.05 divided by 50) would be considered statistically significant for secondary analyses. We used inverse probability weighting (IPW) approach to address potential selection bias due to missing observations (see supplementary materials).

For qualitative analysis on free text responses, we used thematic analysis to identify themes relating to the perceptions of why exposure to the policy could make participants' current ED symptoms better or worse. Thematic analysis is the process of identifying, analyzing, and reporting patterns or themes within qualitative data (Braun & Clarke, 2006). Two authors (MP and LW) independently familiarized themselves with the individual quotes, generated some initial codes, and searched for suitable themes. This process was followed by reviewing and discussing discrepancies in the themes and sub‐themes between both authors. Furthermore, they together defined and named the themes and sub‐themes. For the final process, both authors compiled extract examples and analyzed the selected extracts.

## 3 RESULTS

### 3.1 Characteristics of the participants

The sample consisted of 583 (46%) participants with an ED diagnosis and 690 (54%) participants without an ED diagnosis but with a diagnosis of another mental health condition (Table 1). Participants were predominantly women, White, and completed a degree‐level qualification. Participants with an ED tended to be younger and had a higher proportion of underweight, but lower proportions of overweight and obesity than those without an ED. Those with an ED scored higher for current ED and mental health symptomatology. Anorexia nervosa (32%), bulimia nervosa (25%), and binge‐ED (34%) were the three main diagnoses ever reported by participants with an ED. In addition, generalized anxiety disorder (76%) and major depressive disorder (79%) were the most common types of ever‐reported other mental health conditions.

### 3.2 Acceptability and perceptions of obesity‐related public health policies

Participants with an ED had a lower level of agreement (i.e., agree, strongly agree) on the implementation of calorie labeling policy than their counterparts without an ED (43% vs. 58%) (Table 2). Half of the participants with an ED (55%) reported that calorie labeling may worsen their ED symptoms. Relative to neutral, participants with an ED (vs. without) were significantly more likely to disagree with the government implementing the policy (RRR = 1.96; 95% CI = 1.23, 3.13) (Table 3). Participants with an ED (vs. without) were significantly less likely to disagree (relative to neutral) that calorie labeling would make them feel anxious. Those with an ED diagnosis (vs. without) were significantly more likely to agree (relative to neutral) that calorie labeling would increase feelings of guilt (RRR = 1.77; 95% CI = 1.16, 2.72) and lead to them being more afraid when eating out (RRR = 2.00; 95% CI = 1.31, 3.07). However, there were also significant tendencies for agreement (relative to neutral) in participants with an ED (vs. without) that calorie labeling would make them feel less guilty (RRR = 2.17; 95% CI = 1.39, 3.40) and less afraid (RRR = 1.72; 95% CI = 1.15, 2.57) when eating out.

**TABLE 2:** Acceptability and perceptions of mandatory calorie labeling on menus in participants who have and have not been diagnosed with an ED.

Items	*n*	Strongly disagree (%)	Disagree (%)	Neutral (%)	Agree (%)	Strongly agree (%)
*Businesses like restaurants, fast food outlets and coffee shops should be required to display the calorie content of their foods on menus and menu boards*.						
ED diagnosis	583	27.79	18.18	10.98	21.10	21.96
No ED diagnosis	690	10.29	15.07	16.23	33.91	24.49
*Calorie information on menus and menu boards will be helpful when deciding what I want to choose when eating out*.						
ED diagnosis	582	25.95	14.95	10.14	24.91	24.05
No ED diagnosis	688	11.34	16.28	9.74	35.61	27.03
*I will feel anxious if I see calorie information on menus and menu boards when eating out*.						
ED diagnosis	583	8.23	16.47	12.69	23.84	38.77
No ED diagnosis	690	22.75	32.61	13.77	19.57	11.30
*Compared to eating out without calorie labeling information*, *calorie labeling will make me feel **more guilty** when eating out*.						
ED diagnosis	583	6.69	12.35	11.15	22.98	46.83
No ED diagnosis	690	10.29	25.94	17.10	25.65	21.01
*Compared to eating out without calorie labeling information, calorie labeling will make me feel **less guilty** when eating out*.						
ED diagnosis	583	38.08	29.16	14.41	13.21	5.15
No ED diagnosis	690	18.41	35.22	28.84	13.91	3.62
*Compared to eating out without calorie labeling*, *calorie labeling will make me feel **more afraid** about eating out*.						
ED diagnosis	583	9.43	22.81	16.12	22.98	28.64
No ED diagnosis	690	24.20	35.94	16.38	17.10	6.38
*Compared to eating out without calorie labeling*, *calorie labeling will make me feel **less afraid** about eating out*.						
ED diagnosis	583	29.33	29.67	20.93	14.92	5.15
No ED diagnosis	689	16.69	29.90	33.38	16.55	3.48
*If a menu without calorie information was also available, I would prefer to use that when eating out*.						
ED diagnosis	582	13.23	19.76	12.71	19.93	34.36
No ED diagnosis	690	12.46	23.77	26.67	22.90	14.20
*If a menu without calorie information was also available, I would feel comfortable asking for it when eating out*.						
ED diagnosis	583	20.24	36.02	14.75	19.38	9.61
No ED diagnosis	690	13.77	31.88	21.45	24.64	8.26

Items	*n*	Much worse (%)	Somewhat worse (%)	No different/unsure (%)	Somewhat better (%)	Much better (%)
*Seeing calorie information on menus or menu boards when eating out will make my **eating disorder symptoms…*** ^a^						
ED diagnosis	578	28.55	26.47	30.62	9.52	4.84
*Seeing calorie information on menus or menu boards when eating out will make my **other mental health symptoms…***						
ED diagnosis	516	23.06	29.65	36.63	7.17	3.49
No ED diagnosis	687	6.99	20.82	60.84	8.73	2.62

Abbreviations: ED diagnosis, participants who have been diagnosed with an ED; *n*, number of participants; No ED diagnosis; participants who have not been diagnosed with an ED.

^a^ This item was only administered to participants who have been diagnosed with an ED.

**TABLE 3:** Analyses examining differences in acceptability and perceptions of mandatory calorie labeling on menus between participants who have and have not been diagnosed with an ED.

Dependent variables	Disagree versus neutral		Agree versus neutral	
	RRR	95% CI	RRR	95% CI
*Businesses like restaurants*, *fast food outlets*, *and coffee shops should be required to display the calorie content of their foods on menus and menu boards* (*n = 915*; *ref =* *No ED diagnosis* )				
ED diagnosis	1.96	1.23, 3.13**	1.09	0.71, 1.69
*Calorie information on menus and menu boards will be helpful when deciding what I want to choose when eating out* (*n = 913*; *ref =* *No ED diagnosis* )				
ED diagnosis	1.10	0.65, 1.85	0.73	0.45, 1.20
*I will feel anxious if I see calorie information on menus and menu boards when eating out* (*n = 915; ref =* *No ED diagnosis* )				
ED diagnosis	0.44	0.28, 0.68***	1.41	0.90, 2.20
*Compared to eating out without calorie labeling information*, *calorie labeling will make me feel **more guilty** when eating out* (*n = 915*; *ref* = *No ED diagnosis* )				
ED diagnosis	0.81	0.51, 1.29	1.77	1.16, 2.72**
*Compared to eating out without calorie labeling information*, *calorie labeling will make me feel **less guilty** when eating out* (*n = 915*; *ref* = *No ED diagnosis* )				
ED diagnosis	2.10	1.14, 3.07***	2.17	1.39, 3.40**
*Compared to eating out without calorie* labeling, *calorie* labeling *will make me feel **more afraid** about eating out* (*n* = *915*; *ref =* *No ED diagnosis* )				
ED diagnosis	0.71	0.47, 1.07	2.00	1.31, 3.07**
*Compared to eating out without calorie* labeling, *calorie* labeling *will make me feel **less afraid** about eating out* (*n* = *914*; *ref* = *No ED diagnosis* )				
ED diagnosis	1.62	1.15, 2.28**	1.72	1.15, 2.57**
*If a menu without calorie information was also available*, *I would prefer to use that when eating out* (*n* = *914*; *ref* = *No ED diagnosis* )				
ED diagnosis	1.96	1.29, 2.99**	2.60	1.71, 3.94***
*If a menu without calorie information was also available*, *I would feel comfortable asking for it when eating out* (*n = 915*; *ref* = *No ED diagnosis* )				
ED diagnosis	1.51	1.01, 2.24*	1.22	0.80, 1.86

Dependent variables	Worse versus neutral		Better versus neutral	
	RRR	95% CI	RRR	95% CI
*Seeing calorie information on menus or menu boards when eating out will make my **other*** ***mental health symptoms*** (*n* = *872*; *ref =* *No ED diagnosis* )				
ED diagnosis	2.59	1.86, 3.59***	1.97	1.27, 3.08**

*Note*: Multinomial logistic regression models were developed for each item of acceptability and perceptions of the policy, adjusting for age, gender, ethnicity, education, tertiles of equivalised household income, and BMI category.

Abbreviations: CI, confidence interval; ED diagnosis, participants who have been diagnosed with an ED; No ED diagnosis, participants who have not been diagnosed with an ED; ref, reference group; RRR, relative risk ratio.

* *p* < .05;

** *p* < .01;

*** *p* < .001.

Participants with an ED (vs. without) were also significantly more likely to both agree and disagree (relative to neutral) that they would prefer to use a menu with calorie labeling when it was available. However, participants with an ED (vs. without) were significantly more likely to disagree (relative to neutral) that they would feel comfortable asking for it when eating out. Furthermore, participants with an ED (vs. without) were significantly more likely to perceive that calorie labeling would make their current mental health symptoms both worse and better (relative to neutral). When ED and mental health symptomatology were adjusted, relative to neutral, having an ED diagnosis (vs. without) was only associated with perceptions of worsening other mental health symptoms at corrected *p* < .001 (see Table S4).

We found no differences in acceptability and perceptions of calorie labeling by the status of current ED symptoms (having current vs. past diagnosis) among participants with an ED (Table S5). Among participants with an ED, no significant associations were found between being diagnosed with any EDs and tendencies of using and asking for a menu with calorie labeling (Table S6). ED diagnoses (e.g., anorexia nervosa, bulimia nervosa) were not statistically significant when associated with perceived worsening ED symptoms at corrected *p* < .001 (Table 4, and see Table S7 for additional adjustment for ED and mental health symptomatology).

**TABLE 4:** Adjusted associations between diagnoses of ED and other mental health conditions and perceived effect of calorie labeling policy on the current symptoms in participants who have been diagnosed with an ED.

Dependent variables	Worse versus neutral		Better versus neutral	
	RRR	95% CI	RRR	95% CI
*Seeing calorie information on menus or menu boards when eating out will make my **eating disorder symptoms*** (*n* = *350*)				
Anorexia nervosa (*yes* vs. *no*)	2.45	1.24, 4.83*	0.72	0.26, 2.00
Bulimia nervosa (*yes* vs. *no*)	2.64	1.39, 5.00**	2.08	0.89, 4.85
Binge‐eating disorder (*yes* vs. *no*)	1.00	0.53, 1.89	1.49	0.67, 3.30
Generalized anxiety disorder (*yes* vs. *no*)	0.83	0.43, 1.58	1.83	0.70, 4.78
Major depressive disorder (*yes* vs. *no*)	0.53	0.27, 1.02	0.33	0.15, 0.73**
*Seeing calorie information on menus or menu boards when eating out will make my **other*** ***mental health symptoms*** (*n* = *349*)				
Anorexia nervosa (*yes* vs. *no*)	1.38	0.75, 2.54	0.46	0.17, 1.24
Bulimia nervosa (*yes* vs. *no)*	1.66	0.93, 2.94	1.46	0.64, 3.36
Binge‐eating disorder (*yes* vs. *no*)	1.07	0.59, 1.95	0.96	0.42, 2.20
Generalized anxiety disorder (*yes* vs. *no*)	1.37	0.76, 2.46	2.42	0.90, 6.46
Major depressive disorder (*yes* vs. *no*)	0.87	0.48, 1.58	0.71	0.32, 1.58

*Note*: Multinomial logistic regression models were developed for each item of acceptability and perceptions of the policy, adjusting for age, gender, ethnicity, education, tertiles of equivalised household income, and BMI category. *p < .05, **p < .01, ***p < .001.

Abbreviations: CI, confidence interval; ref, reference group; RRR, relative risk ratio.

The level of agreement on the policy of banning advertisements of unhealthy food and drinks was slightly lower in participants with versus without an ED (49% vs. 55%) (Table S8), although regression analyses indicated no significant differences in the likelihood of acceptability and most perceptions of that policy between these two groups (Table S9). However, relative to neutral, participants with an ED (vs. without) were significantly less likely to disagree that the policy would not bring benefits to children's health than their counterparts without an ED (RRR = 0.69, 95% CI = 0.48, 0.98). For the policy related to banning “buy one get one free” deals for unhealthy food and drinks, we found similar levels of agreement between participants with and without an ED (29% vs. 30%) (Table S10). No differences in acceptability and perceptions of this policy were observed across participant groups (Table S11). Findings on the acceptability and perceived harm of all the policies above were largely consistent when IPW approach was used to address missing observations (Tables S12–S15).

### 3.3 Qualitative findings

As perceptions of perceived harm due to policies differed between participants with and without an ED for calorie labeling, we focused on free‐text responses relating to perceived effects of this policy on current ED symptoms. Qualitative findings indicated perceived potential harm and benefits of calorie labeling in participants with an ED (Table 5).

**TABLE 5:** Thematic analysis results: Themes related to calorie labeling on menus in participants with an ED.

Themes	Sub‐themes	Quotes
Responses to: Seeing calorie information on menus or menu boards when eating out will make my eating disorder symptoms **“somewhat worse”** or **“much worse”**		
1. Hyper fixation and gateway to relapse	Hyper fixation on calories	“I become hyperaware of the idea of the calories, I imagine my body ballooning up, I feel dirty.” (*ID 100631, has ever been diagnosed with binge‐eating disorder and OSFED)* “Part of my disorder is obsessing over calorie content and seeing them on menus would make it much harder not to focus on it.” (*ID 101145, has ever been diagnosed with anorexia nervosa and UFED*)
	Relapse in ED recovery	“I am on a binge restrict cycle with my eating disorder. Calorie information feeds into this as it facilitates restriction, but also if I have a calorie expensive meal then it will trigger a binging episode as the damage is already done.” (*ID 100612, has ever been diagnosed with bulimia nervosa and binge‐eating disorder*) “…seeing the [calorie] info has often triggered days‐long spirals for me.” (*ID 100882*, *specific eating disorder diagnosis was not reported*)
2. Negative effects on mood	Feeling guilty and anxiety	“… I begin to feel guilty for the calories I was about to consume, and this can lead to me restricting my diet for a few days.” (*ID 100030, has ever been diagnosed with bulimia nervosa*) “I have only been able to recover […] by avoiding calorie counts as much as possible. Having this information shoved in my face makes the guilt and anxiety around calories return.” (*ID 100895, has ever been diagnosed with UFED*)
	Decreasing social enjoyment	“Eating out is a fun social experience. It is not something I do every day and I do not want this spoiling for me by having to worry about calories” (*ID 100760, has ever been diagnosed with anorexia nervosa*) “Since they started listing calories on menus I've not been able to eat out as everything seems too high in calories to be able to eat anything. […] I just haven't been able to choose anything, so have had nothing to eat.” (*ID 101133, has ever been diagnosed with anorexia nervosa*)
Responses to: Seeing calorie information on menus or menu boards when eating out will make my eating disorder symptoms **“somewhat better”** or **“much better”**		
3. Calorie counting and control	Calorie counting	“Counting calories is a way to be able to eat ‘unsafe’ foods healthily by including them in my calorie count for the day […]. It is when I completely restrict unsafe foods because I'm scared of them, that I end up bingeing” (*ID 100214, has ever been diagnosed with OSFED*) “I would be able to plan what I am eating later on in the day if I know how many calories I have consumed for that meal, in order to stick into calorie deficit” (*ID 100747, has ever been diagnosed with a binge‐eating disorder*)
	Control of overeating	“I feel more in control and can make decisions knowing the calories in the items.” (*ID 101013, has ever been diagnosed with avoidant/restrictive food intake disorder*) “It will act as a reminder and may stop me from going overboard in the moment.” (*ID 101103*, *has ever been diagnosed with a binge‐eating disorder*)
4. Feeling informed and reassured	Feeling informed	“I would feel more informed about what I was eating. I have a rough idea of calorie amounts but I genuinely would feel better being able to make healthier informed choices.” (*ID 100238, specific eating disorder diagnosis was not reported*) “It really helps me put food into context and feel more informed about what I am putting in my body.” (*ID 100672, specific eating disorder diagnosis was not reported*)
	Reassurance and reduced anxiety	“There will be no worry or guilt if I know what the content of what I have had to eat and therefore make it less likely for me to relapse because I will feel reassured I have already made the right choice.” (*ID 100270*, *has ever been diagnosed with bulimia nervosa*) “Most of my anxiety from eating outcomes from not knowing what is in the food… This then often leads to making poor decisions around food… and/or overestimating the amount of calories in a meal, which then means restricting more during other meals. I would much rather be empowered to make conscious decisions about my food intake.” (*ID 100958, has ever been diagnosed with bulimia nervosa and binge‐eating disorder*)

Abbreviations: OSFED, Other specified feeding or eating disorder; UFED, Unspecified feeding or eating disorder.

#### 3.3.1 Negative effects

Theme 1: *Hyper fixation and gateway to relapse* was an underlying theme for why participants perceived potential personal harm of calorie labeling. Participants felt that calorie labeling would make them have a *hyper fixation* or be obsessive about calorie labeling, which was suggested by some participants to be a potential *gateway to relaps*e.

Theme 2: A common theme was that calorie labeling may have *negative effects on mood*, prompting feelings of guilt and anxiety. In addition, while eating out was perceived as social pleasure, seeing calorie labeling would decrease enjoyment and lower mood.

#### 3.3.2 Positive effects

Theme 3: *Calorie counting and control* appeared as a self‐perceived positive effect of calorie labeling on menus. Participants reported that calorie labeling may help them to track calories when eating out. Calorie information would also allow participants to include “unsafe” foods in a calorie count for the day and feel in control.

Theme 4: Some participants reported that they would *feel informed and reassured* when calorie labeling was available when eating out. Participants would feel better and less guilty because of being able to make healthier informed choices on what they put in their bodies.

## 4 DISCUSSION

This study examined the acceptability and perceptions of three UK obesity‐related public health policies in people with an ED and other mental health conditions. Both participants with and without an ED equally supported the implementation of obesity policies on restricted marketing and price promotions of unhealthy food and drinks. No significant differences in perceived harm of these policies were observed between participants with versus without an ED diagnosis. However, acceptability and perceived harm for calorie labeling differed between participants with and without an ED.

We found that having an ED diagnosis (vs. without) was associated with a greater likelihood of disagreement with the implementation of mandatory calorie labeling and perceptions of its negative effects (e.g., feeling anxious). Although moderate‐to‐high levels of support have been reported for the calorie labeling policy in the general population across different age groups (Beeken & Wardle, 2013; Bhawra et al., 2018; Gollust et al., 2014), less support tends to be expressed by people with disordered eating (Royal College of Psychiatrists, 2022a). Our qualitative findings showed that potential relapse in ED recovery and hyper fixation on calories were reasons for the disagreement with this policy in people with an ED. This aligns with previous investigations suggesting that people with disordered eating tendencies are more likely to use menu labels to choose lower‐calorie options (Christoph et al., 2018; Larson et al., 2018). In an online study, participants scored higher for anorexia nervosa and bulimia nervosa symptomatology would opt to consume fewer calories, and those with higher binge‐ED symptomatology more calories, when provided with hypothetical calorie information (Haynos & Roberto, 2017). However, a pre‐post experimental study in a university cafeteria by Lillico et al. (2015) found no adverse effects of calorie labeling on emotional states and unhealthy weight‐related behaviors such as binging or restricting calories in participants with a risk of ED.

In this study, the perceptions of calorie labeling did not differ by the status of ED diagnosis (past vs. current diagnosis). Therefore, this policy may impact both participants with active ED and those who were diagnosed in the past with minimal current symptomatology to a similar extent. In addition, no specific ED was statistically significantly associated with perceived harm of calorie labeling on current ED symptoms more so than any other specific ED, at corrected *p* < .001. However, findings should be interpreted with some caution as analyses may have been limited in power due to the correction for multiple comparisons used.

In this study, calorie labeling was not perceived negatively by all participants with an ED. Participants with an ED (vs. without) were also more likely to agree (relative to neutral) that they would feel less guilty and less afraid when eating out with calorie labeling information. A previous investigation found that people with an ED are also largely in favor of the calorie labeling policy (Roberto et al., 2013). Qualitative data indicated that the reasons for perceived benefits of calorie labeling included helping keep track of calories and control overeating as individuals with ED may unknowingly overestimate their food intake (Bartholome et al., 2013; Sysko et al., 2005). In addition, it was expressed that calorie labeling would allow participants to make more informed choices leading to increased feelings of reassurance and reduced anxiety. However, further investigation is needed to understand whether calorie labeling has genuine benefits for the health and well‐being of some individuals with an ED. Whilst calorie counting may increase control over food intake and temporarily reduce guilt and anxiety for those with an ED, this may also encourage restriction in the long term. Given the associations between calorie counting and increased ED severity (Romano et al., 2018; Simpson & Mazzeo, 2017), some caution should be also taken when interpreting these findings.

These findings have important implications for policy. As calorie labeling may encourage healthier food choices (Robinson et al., 2023; Sinclair et al., 2014) and has received support from the public (Beeken & Wardle, 2013; Bhawra et al., 2018; Gollust et al., 2014) and people with an ED (Roberto et al., 2013) (including some participants with an ED and other mental health conditions in this study), ensuring people have choices of menus with and without calorie labeling will be important to accommodate all needs. In food outlets already required by law to include calorie labeling on menus, a menu without calorie labeling available to request in advance, or in a way that mitigates individuals' anxiety in asking for this information may be beneficial. Further research is warranted to understand the impacts of calorie labeling and how to mitigate its potentially harmful impacts in those with an ED.

### 4.1 Study strengths and limitations

This study is the first that investigated the acceptability and perceptions of three UK public health policies to address obesity in people with an ED and other mental health conditions. Findings could inform on the acceptability of calorie labeling and measures that may mitigate negative impacts on people with an ED both in the United Kingdom and internationally. Though, the generalisability of these results to other countries should not be assumed and we encourage assessments of calorie labeling acceptability and impacts in countries currently with or planning to implement mandatory calorie labeling policy, including the United States (Cleveland et al., 2018), Canada (Goodman et al., 2018), and Australia (Wellard‐Cole et al., 2018).

Findings of this study focused on differences in perceived harm by self‐reported ED status and did not examine causal influences of obesity policies (e.g., calorie labeling) on ED symptoms. Other limitations of this study included the sampling method used which resulted in a predominantly White, well‐educated, and female participant sample. Given that ED is typically more common among lower socioeconomic status and minority groups (Burke et al., 2022), further research may benefit from exploring acceptability and perceptions of calorie labeling among these populations. In addition, ED diagnosis relied upon self‐report doctor diagnosis and may not be fully accurate. However, self‐reported ED diagnosis has been used in some previous studies (Pedram et al., 2021; Termorshuizen et al., 2020) and was found to be at least as good as other available instruments for population screening (Keski‐Rahkonen et al., 2006). While studies on the accuracy of self‐reported ED diagnosis are limited, self‐reports of other mental health conditions, such as depression have been found to not differ statistically from formal psychiatric diagnosis (Sanchez‐Villegas et al., 2008; Santos et al., 2021). We also note that in a small number of instances, findings were not considered statistically significant when Bonferroni corrected (e.g., the associations between specific ED diagnoses, such as anorexia nervosa, and perceived harm of calorie labeling on current ED symptoms). Because this level of stringent statistical correction reduces statistical power and can inflate type II error (Nakagawa, 2004; Sedgwick, 2012), caution in the interpretation of these non‐significant findings is needed. Moreover, due to the smaller sample size of people with an ED, analyses examining acceptability and reported impact of calorie labelling by symptom severity and socioeconomic status will benefit from being replicated in larger and more representative samples. Furthermore, the current study did not measure participants' current behaviors toward counting calories. Future research would benefit from assessing the potential differing impacts of calorie labeling policies on people who count calories versus people who do not in both people with and without an ED. Additionally, this research area would benefit from future studies examining real‐world behavior and directly measured impacts from exposure to calorie labeling in people with an ED, such as, if calorie labeling triggers people with an ED to count calories and if exposure to mandatory calorie labeling increases the risk of an ED or disordered eating. It would also be beneficial for future research to have a specific focus on young adults who are often at a higher risk of developing disordered eating (Sharps et al., 2022) and are more frequent consumers of food purchased out of the home (Adams et al., 2015).

## 5 CONCLUSION

This survey study of participants with an ED and other mental health conditions found that whilst obesity policies on price and marketing of unhealthy food and drinks were generally deemed acceptable and unlikely to worsen mental health, calorie labeling was less well supported and was reported to have more negative impacts on people with an ED compared to those with other mental health conditions. Further research is warranted to explore the impacts of calorie labeling policy in people with an ED.

## AUTHOR CONTRIBUTIONS

**I Gusti Ngurah Edi Putra:** Conceptualization; data curation; formal analysis; funding acquisition; methodology; project administration; visualization; writing – original draft. **Megan Polden:** Conceptualization; formal analysis; methodology; writing – original draft. **Lettie Wareing:** Formal analysis; methodology; writing – original draft. **Eric Robinson:** Conceptualization; funding acquisition; methodology; supervision; writing – review and editing.

## CONFLICT OF INTEREST STATEMENT

The authors declare that there are no conflict of interest.

### OPEN RESEARCH BADGES

This article has earned an Open Data badge for making publicly available the digitally‐shareable data necessary to reproduce the reported results. The data is available at [https://doi.org/10.17605/OSF.IO/UTCR7].

## Supporting information


**Data S1:** Supporting information

## Data Availability

The data that support the findings of this study are openly available at https://doi.org/10.17605/OSF.IO/UTCR7.
