# Supplementary material for: Acceptability and perceived harm of calorie labeling and other obesity policies: A cross‐sectional survey study of UK adults with eating disorders and other mental health conditions
Source: Int J Eat Disord. 2023 Jul 28;56(11):2049–61. doi: 10.1002/eat.24031 (PMC10947290; doi:10.1002/eat.24031)
Supplement: Supplementary file 1 — Data S1: Supporting information [file EAT-56-2049-s001.pdf]

Supplementary materials

Sample size calculation

We calculated a minimum sample size using G*POWER 3.1.9.7 with Z-test selected for the test family and logistic regression for the statistical test and information in the following table was also supplied (Demidenko, 2007; Faul et al., 2009). To detect statistically small differences in the proportion (10%) of agreement or disagreement between those with vs. without an eating disorder (ED), a minimum sample size of 865 participants was required for logistic regression analysis.

Table S1. Information inputted for sample size calculation

**Required information**		**Description**
Tail(s)	Two	Testing a deviation from the Ho in both directions
Pr(Y=1|X=1) H0	.50	An anticipated proportion of people without ED (with other mental health conditions) disagree with the policy
Pr(Y=1|X=1) H1	.60	An anticipated proportion of people with ED disagree with the policy at least 10% higher than people without ED
α err prob	.05	Significance level (type I error)
Power (1-β err prob)	.80	Power (1 – type II error)
R^2 other X	.10	An expected R-squared value between the main predictor variable and other covariates
X distribution	Binomial	Distribution of the predictor variable (binomial: with vs. without ED)
X parm π	.50	A balanced design with equal sample frequencies

Validity and reliability of psychometric instruments

A 7-item Eating Disorder Examination Questionnaire (EDE-Q7)

We used exploratory factor analysis (EFA) to assess the validity of EDE-Q7 by identifying relationships among indicators or items. Cronbach's Alpha was used to determine the internal consistency reliability (Fenn et al., 2020). When all seven items were included together, findings from EFA indicated that two factors had Eigenvalue > 1.0 (Factor 1 with Eigenvalue = 3.23 and Factor 2 with Eigenvalue = 2.58). Varimax rotation was then used to simplify and clarify data structure and identify separate factors (Osborne, 2015). We found four EDE-Q7 items that form the shape/weight overvaluation and body dissatisfaction subscales had factor loadings ranging from .86 to .89 and these items constituted Factor 1. In addition, the other three items that form the dietary restraint subscale defined Factor 2 and had factor loadings ranging from .88 to .90. Findings from EFA also indicated no crossloadings as no items that loaded at .32 or higher on both factors (Costello & Osborne, 2005). We calculated an average

score of four items that developed Factor 1 (the shape/weight overvaluation and body dissatisfaction subscales) to indicate ED symptomatology. This four-item measure was found to have very good internal consistency with Cronbach's Alpha = .93 (see Table S2).

Table S2. Validity and reliability of EDE-Q7

**Factor 2: Dietary restraint**<br>"On how many of the past 28 days ……"	**Factor loadings**	**Cronbach's Alpha**
1. "Have you been consciously trying to limit the amount of food you eat to influence your shape or weight?"	.90	.90
2. "Have you attempted to avoid eating any foods which you like in order to influence your shape or weight?"	.88	
3. "Have you attempted to follow definite rules regarding your eating in order to influence your shape or weight; for example, a calorie limit, a set amount of food, or rules about what or when you should eat?"	.88	
**Factor 1: Shape/weight overvaluation (items 4 and 5)**<br>**Body dissatisfaction (items 6 and 7)**<br>"On how many over the past 28 days ……"	**Factor loadings**	**Cronbach's Alpha**
4. "Has your weight influenced how you think about (judge) yourself as a person?"	.86	.93
5. "Has your shape influenced how you think about (judge) yourself as a person?"	.87	
6. "How dissatisfied have you felt about your weight?"	.89	
7. "How dissatisfied have you felt about your shape?"	.89	

A 4-item Patient Health Questionnaire (PHQ-4)

EFA was also used to evaluate the validity and Cronbach's Alpha was used to determine the reliability of PHQ-4 (Fenn et al., 2020). Findings from EFA showed that only one factor was found with Eigenvalue > 1.0, and therefore, all the items can be aggregated together. All items also had high factor loadings (≥ .84). PHQ-4 also had good internal consistency (.88) (see Table S3).

Table S3. Validity and reliability of PHQ-4

**PHQ-4**<br>"Over the last 2 weeks, how often have you been bothered by the following problems?"	**Factor loadings**	**Cronbach's Alpha**
1. "Feeling nervous, anxious, or on edge"	.85	.88
2. "Not being able to stop or control worrying"	.87	
3. "Feeling down, depressed, or hopeless"	.87	
4. "Little interest or pleasure in doing things"	.84	

Robustness analysis: inverse probability weighting (IPW)

IPW approach was used to address missing values and potential selection bias due to some characteristics that may be associated with sample retention (Chesnaye et al., 2022; Mansournia & Altman, 2016). Based on the observed data, we found that increasing age and degree-level qualification completion were associated with having complete observations. Women and other genders were less likely to have complete observations compared to men. Using a logistic regression model, we estimated the probability of being retained in the analytical sample size (i.e., having complete vs. incomplete observations) based on sociodemographic characteristics associated with missingness (age, gender, educational level). We then calculated sample weights as the inverse probability of retention. We applied these weights in the multinomial logistic regression analysis to provide robust standard errors after taking into account differences in participants' characteristics associated with missingness.

References

Chesnaye, N. C., Stel, V. S., Tripepi, G., Dekker, F. W., Fu, E. L., Zoccali, C., & Jager, K. J. (2022). An introduction to inverse probability of treatment weighting in observational research. *Clinical Kidney Journal, 15*(1), 14-20. https://doi.org/10.1093/ckj/sfab158

Costello, A. B., & Osborne, J. (2005). Best practices in exploratory factor analysis: four recommendations for getting the most from your analysis. *Practical Assessment, Research, and Evaluation, 20*. https://doi.org/https://doi.org/10.7275/jyj1-4868

Demidenko, E. (2007). Sample size determination for logistic regression revisited. *Statistics in Medicine, 26*(18), 3385-3397. https://doi.org/https://doi.org/10.1002/sim.2771

Faul, F., Erdfelder, E., Buchner, A., & Lang, A.-G. (2009). Statistical power analyses using G*Power 3.1: Tests for correlation and regression analyses. *Behavior Research Methods, 41*(4), 1149-1160. https://doi.org/10.3758/BRM.41.4.1149

Fenn, J., Tan, C.-S., & George, S. (2020). Development, validation and translation of psychological tests. *BJPsych Advances, 26*(5), 306-315. https://doi.org/10.1192/bja.2020.33

Mansournia, M. A., & Altman, D. G. (2016). Inverse probability weighting. *BMJ, 352*, i189. https://doi.org/10.1136/bmj.i189

Osborne, J. W. (2015). What is Rotating in Exploratory Factor Analysis? *Practical Assessment, Research, and Evaluation, 20*. https://doi.org/https://doi.org/10.7275/hb2g-m060

Additional tables for the results section

Table S4. Differences in acceptability and perceptions of mandatory calorie labelling on menus between participants who have and have not been diagnosed with an ED (models include additional adjustments for ED and mental health symptomology)

Dependent variables	Disagree vs. neutral		Agree vs. neutral	
	RRR	95% CI	RRR	95% CI
Businesses like restaurants, fast food outlets and coffee shops should be required to display the calorie content of their foods on menus and menu boards (n = 903; ref = No ED diagnosis)				
ED diagnosis	1.85	1.13, 3.03*	0.92	0.59, 1.46
Calorie information on menus and menu boards will be helpful when deciding what I want to choose when eating out (n = 901; ref = No ED diagnosis)				
ED diagnosis	1.00	0.57, 1.75	0.58	0.34, 0.99*
I will feel anxious if I see calorie information on menus and menu boards when eating out (n = 903; ref = No ED diagnosis)				
ED diagnosis	0.46	0.29, 0.74**	1.18	0.74, 1.89
Compared to eating out without calorie labelling information, calorie labelling will make me feel ***more guilty*** *when eating out (n = 903; ref = No ED diagnosis)*				
ED diagnosis	0.80	0.49, 1.29	1.48	0.95, 2.32
Compared to eating out without calorie labelling information, calorie labelling will make me feel ***less guilty*** *when eating out (n = 903; ref = No ED diagnosis)*				
ED diagnosis	1.93	1.30, 2.86**	1.79	1.12, 2.85*
Compared to eating out without calorie labelling, calorie labelling will make me feel ***more afraid*** *about eating out (n = 903; ref = No ED diagnosis)*				
ED diagnosis	0.77	0.51, 1.17	1.71	1.10, 2.67*
Compared to eating out without calorie labelling, calorie labelling will make me feel ***less afraid*** *about eating out (n = 902; ref = No ED diagnosis)*				
ED diagnosis	1.55	1.08, 2.21*	1.55	1.02, 2.36*
If a menu without calorie information was also available, I would prefer to use that when eating out (n = 902 ref = No ED diagnosis)				
ED diagnosis	1.55	1.00, 2.41*	2.09	1.35, 3.22**
If a menu without calorie information was also available, I would feel comfortable asking for it when eating out (n = 903; ref = No ED diagnosis)				
ED diagnosis	1.24	0.82, 1.88	1.19	0.77, 1.86
**Dependent variables**	**Worse vs. neutral**		**Better vs. neutral**	
	**RRR**	**95% CI**	**RRR**	**95% CI**
Seeing calorie information on menus or menu boards when eating out will make my ***other mental health symptoms*** *(n = 860; ref = No ED diagnosis)*				
ED diagnosis	1.97	1.39, 2.78***	1.52	0.95, 2.43

*p < .05; **p < .01; ***p < .001

RRR = relative risk ratio; CI = confidence interval; ref = reference group; ED diagnosis = participants who have been diagnosed with an ED; No ED diagnosis= participants who have not been diagnosed with an ED

Multinomial logistic regression models were developed for each item of acceptability and perceptions of the policy, adjusting for age, gender, ethnicity, education, tertiles of equivalised household income, BMI category, ED symptoms from EDE-Q7 (shape/weight overvaluation, body dissatisfaction), and mental health symptoms from PHQ-4.

Table S5. Adjusted associations between the status of current ED and opinions on mandatory calorie labelling policy in participants who have been diagnosed with an ED

Dependent variables	Disagree vs. neutral		Agree vs. neutral	
	**RRR**	**95% CI**	**RRR**	**95% CI**
Businesses like restaurants, fast food outlets and coffee shops should be required to display the calorie content of their foods on menus and menu boards (n = 385; ref = Past ED diagnosis)				
Current ED diagnosis	0.55	0.25, 1.19	0.78	0.38, 1.62
Calorie information on menus and menu boards will be helpful when deciding what I want to choose when eating out (n = 385; ref = Past ED diagnosis)				
Current ED diagnosis	1.27	0.53, 3.02	1.89	0.83, 4.31
I will feel anxious if I see calorie information on menus and menu boards when eating out (n = 385; ref = Past ED diagnosis)				
Current ED diagnosis	1.25	0.59, 2.67	1.15	0.58, 2.29
Compared to eating out without calorie labelling information, calorie labelling will make me feel ***more guilty*** *when eating out (n = 385; ref = Past ED diagnosis)*				
Current ED diagnosis	1.38	0.60, 3.18	1.08	0.51, 2.29
Compared to eating out without calorie labelling information, calorie labelling will make me feel ***less guilty*** *when eating out (n = 385; ref = Past ED diagnosis)*				
Current ED diagnosis	1.38	0.68, 2.80	1.86	0.84, 4.13
Compared to eating out without calorie labelling, calorie labelling will make me feel ***more afraid*** *about eating out (n = 385; ref = Past ED diagnosis)*				
Current ED diagnosis	1.42	0.69, 2.90	1.50	0.76, 2.98
Compared to eating out without calorie labelling, calorie labelling will make me feel ***less afraid*** *about eating out (n = 385; ref = Past ED diagnosis)*				
Current ED diagnosis	1.28	0.71, 2.34	1.44	0.72, 2.89
If a menu without calorie information was also available, I would prefer to use that when eating out (n = 384; ref = Past ED diagnosis)				
Current ED diagnosis	0.92	0.43, 1.97	1.26	0.60, 2.65
If a menu without calorie information was also available, I would feel comfortable asking for it when eating out (n = 385; ref = Past ED diagnosis)				
Current ED diagnosis	1.13	0.57, 2.24	0.97	0.46, 2.04
**Dependent variables**	**Worse vs. neutral**		**Better vs. neutral**	
	**RRR**	**95% CI**	**RRR**	**95% CI**
Seeing calorie information on menus or menu boards when eating out will make my ***eating disorder symptoms*** *(n = 385; ref = Past ED diagnosis)*				
Current ED diagnosis	0.84	0.49, 1.43	0.93	0.48, 1.80
Seeing calorie information on menus or menu boards when eating out will make my ***other mental health symptoms*** *(n = 345; ref = Past ED diagnosis)*				
Current ED diagnosis	1.03	0.61, 1.74	1.28	0.62, 2.67

*p < .05; **p < .01; ***p < .001

RRR = relative risk ratio; CI = confidence interval; ref = reference group; past ED diagnosis = “Past ED and no current symptoms” + “Past ED and some lingering symptoms”; current ED diagnosis = “Currently ED and experience symptoms”

Multinomial logistic regression models were developed for each item of acceptability and perceptions of the policy, adjusting for age, gender, ethnicity, education, tertiles of equivalised household income, and BMI category.

Table S6. Adjusted associations between diagnoses of ED and other mental health conditions and opinions on the use of calorie information in participants who have been diagnosed with an ED

Dependent variables	Disagree vs. neutral		Agree vs. neutral	
	**RRR**	**95% CI**	**RRR**	**95% CI**
If a menu without calorie information was also available, I would prefer to use that when eating out (n = 350)				
Anorexia nervosa *(yes vs. no)*	1.33	0.50, 3.54	1.48	0.58, 3.80
Bulimia nervosa (*yes vs. no)*	1.21	0.48, 3.03	1.67	0.69, 4.03
Binge-eating disorder *(yes vs. no)*	1.50	0.61, 3.72	1.37	0.57, 3.32
Generalized anxiety disorder *(yes vs. no)*	2.35	0.99, 5.58	1.40	0.63, 3.15
Major depressive disorder *(yes vs. no)*	0.56	0.21, 1.51	0.45	0.17, 1.19
If a menu without calorie information was also available, I would feel comfortable asking for it when eating out (n = 351)				
Anorexia nervosa *(yes vs. no)*	0.72	0.32, 1.62	0.56	0.23, 1.38
Bulimia nervosa (*yes vs. no)*	0.84	0.38, 1.88	0.97	0.41, 2.29
Binge-eating disorder *(yes vs. no)*	0.79	0.35, 1.78	0.94	0.39, 2.26
Generalized anxiety disorder *(yes vs. no)*	1.43	0.64, 3.23	1.15	0.48, 2.72
Major depressive disorder *(yes vs. no)*	1.06	0.47, 2.37	0.91	0.39, 2.15

*p < .05; ** p <.01; *** p <.001

RRR = relative risk ratio; CI = confidence interval; ref = reference group

Multinomial logistic regression models were developed for each item of acceptability and perceptions of the policy, adjusting for age, gender, ethnicity, education, tertiles of equivalised household income, and BMI category.

Table S7. Adjusted associations between diagnoses of ED and other mental health conditions and perceived effect of calorie labelling policy on the current symptoms among participants who have been diagnosed with an ED (models include additional adjustments for ED and mental health symptomology)

**Dependent variables**	**Worse vs. neutral**		**Better vs. neutral**	
	**RRR**	**95% CI**	**RRR**	**95% CI**
Seeing calorie information on menus or menu boards when eating out will make my ***eating disorder symptoms*** *(n = 344)*				
Anorexia nervosa *(yes vs. no)*	2.31	1.16, 4.61*	0.65	0.23, 1.86
Bulimia nervosa (*yes vs. no)*	2.56	1.33, 4.93**	1.82	0.77, 4.31
Binge-eating disorder *(yes vs. no)*	0.96	0.49, 1.85	1.30	0.57, 2.94
Generalized anxiety disorder *(yes vs. no)*	0.78	0.40, 1.52	1.65	0.62, 4.41
Major depressive disorder *(yes vs. no)*	0.49	0.24, 0.97*	0.29	0.13, 0.66**
Seeing calorie information on menus or menu boards when eating out will make my ***other mental health symptoms*** *(n = 343)*				
Anorexia nervosa *(yes vs. no)*	1.25	0.67, 2.35	0.42	0.15, 1.17
Bulimia nervosa (*yes vs. no)*	1.50	0.83, 2.72	1.25	0.53, 2.92
Binge-eating disorder *(yes vs. no)*	0.95	0.51, 1.78	0.80	0.34, 1.87
Generalized anxiety disorder *(yes vs. no)*	1.27	0.69, 2.34	2.17	0.80, 5.93
Major depressive disorder *(yes vs. no)*	0.82	0.44, 1.51	0.63	0.28, 1.45

*p < .05; **p < .01; ***p < .001

RRR = relative risk ratio; CI = confidence interval; ref = reference group

Multinomial logistic regression models were developed for each item of acceptability and perceptions of the policy, adjusting for age, gender, ethnicity, education, tertiles of equivalised household income, BMI category, ED symptoms from EDE-Q7 (shape/weight overvaluation, body dissatisfaction), and mental health symptoms from PHQ-4.

Table S8. Acceptability and perceptions of banning advertisements of unhealthy food and drinks online and before 9 pm on TV in participants who have and have not been diagnosed with an ED

Items	n	Strongly disagree (%)	Disagree (%)	Neutral (%)	Agree (%)	Strongly agree (%)
Marketing and advertising of unhealthy foods and drinks (i.e., high fat, salt and/or sugar products) should be banned online and before 9 pm on TV.						
ED diagnosis	579	6.56	19.86	25.04	29.02	19.52
No ED diagnosis	688	6.83	15.99	22.53	34.74	19.91
A ban on marketing and advertising of unhealthy foods and drinks (i.e., high fat, salt and/or sugar products) online and before 9 pm on TV will be helpful to prevent children from seeing more unhealthy food and drink advertisements.						
ED diagnosis	582	6.19	15.64	13.57	36.08	28.52
No ED diagnosis	689	3.34	13.79	11.90	43.25	27.72
A ban on marketing and advertising of unhealthy foods and drinks (i.e., high fat, salt and/or sugar products) online and before 9 pm on TV will not provide benefits to children's health.						
ED diagnosis	581	11.53	36.14	24.96	18.93	8.43
No ED diagnosis	689	10.74	44.70	21.77	17.13	5.66
**Items**	**n**	**Much worse (%)**	**Somewhat worse (%)**	**No different/ unsure (%)**	**Somewhat better (%)**	**Much better (%)**
A ban on marketing and advertising of unhealthy foods and drinks (i.e., high fat, salt and/or sugar products) online and before 9 pm on TV will make my ***eating disorder symptoms…****						
ED diagnosis	577	2.08	3.81	78.86	11.27	3.99
A ban on marketing and advertising of unhealthy foods and drinks (i.e., high fat, salt and/or sugar products) online and before 9 pm on TV will make my ***other mental health symptoms…***						
ED diagnosis	517	1.16	2.13	83.95	9.86	2.90
No ED diagnosis	688	0.58	1.74	85.61	9.16	2.91

*This item was only administered to participants who have been diagnosed with an ED

n = number of participants; % = percentage; ED diagnosis= participants who have been diagnosed with an ED; No ED diagnosis= participants who have not been diagnosed with an ED

Table S9. Differences in acceptability and perceptions of banning advertisements of unhealthy food and drinks online and before 9 pm on TV between participants who have and have not been diagnosed with an ED

Dependent variables	Disagree vs. neutral		Agree vs. neutral	
	**RRR**	**95% CI**	**RRR**	**95% CI**
Marketing and advertising of unhealthy foods and drinks (i.e., high fat, salt and/or sugar products) should be banned online and before 9 pm on TV (n = 915; ref = No ED diagnosis)				
ED diagnosis	1.02	0.68, 1.55	0.88	0.62, 1.25
A ban on marketing and advertising of unhealthy foods and drinks (i.e., high fat, salt and/or sugar products) online and before 9 pm on TV will be helpful to prevent children from seeing more unhealthy food and drink advertisements (n = 915 ref = No ED diagnosis)				
ED diagnosis	0.88	0.53, 1.49	0.68	0.44, 1.05
A ban on marketing and advertising of unhealthy foods and drinks (i.e., high fat, salt and/or sugar products) online and before 9 pm on TV will not provide benefits to children's health (n = 914; ref = No ED diagnosis)				
ED diagnosis	0.69	0.48, 0.98*[a]	0.82	0.54, 1.24
**Dependent variables**	**Worse vs. neutral**		**Better vs. neutral**	
	**RRR**	**95% CI**	**RRR**	**95% CI**
A ban on marketing and advertising of unhealthy foods and drinks (i.e., high fat, salt and/or sugar products) online and before 9 pm on TV will make my ***other mental health symptoms*** *(n = 873; ref = No ED diagnosis)*				
ED diagnosis	1.71	0.65, 4.51	1.32	0.87, 2.02

*p < .05; **p < .01; ***p < .001

RRR = relative risk ratio; CI = confidence interval; ref = reference group; ED diagnosis = participants who have been diagnosed with an ED; No ED diagnosis = participants who have not been diagnosed with an ED

Multinomial logistic regression models were developed for each item of acceptability and perceptions of the policy, adjusting for age, gender, ethnicity, education, tertiles of equivalised household income, and BMI category.

[a]The association remained significant at p < .01 when ED symptoms from EDE-Q7 (shape/weight overvaluation, body dissatisfaction) and mental health symptoms from PHQ-4 were also adjusted into the model (RRR = 0.60; 95% CI = 0.42, 0.88; p = .009; n = 902).

Table S10. Acceptability and perceptions of banning "buy one get one free" deals for unhealthy food and drinks in participants who have and have not been diagnosed with an ED

Items	n	Strongly disagree (%)	Disagree (%)	Neutral (%)	Agree (%)	Strongly agree (%)
"Buy one get one free" deals for unhealthy foods and drinks (i.e., high fat, salt and/or sugar products) should be banned.						
ED diagnosis	581	17.90	31.50	21.86	18.07	10.67
No ED diagnosis	690	17.83	32.46	19.71	18.55	11.45
A ban on "buy one get one free" deals for unhealthy foods and drinks (i.e., high fat, salt and/or sugar products) would be helpful to prevent from buying more unhealthy foods and drinks.						
ED diagnosis	580	11.90	22.59	18.10	33.79	13.62
No ED diagnosis	690	10.14	24.35	17.25	34.06	14.20
A ban on "buy one get one free" deals for unhealthy foods and drinks (i.e., high fat, salt and/or sugar products) will not provide benefits to people's health.						
ED diagnosis	581	9.29	32.19	20.31	25.99	12.22
No ED diagnosis	690	7.68	37.68	21.16	25.51	7.97
**Items**	**n**	**Much worse (%)**	**Somewhat worse (%)**	**No different/ unsure (%)**	**Somewhat better (%)**	**Much better (%)**
A ban on "buy one get one free" deals for unhealthy foods and drinks (i.e., high fat, salt and/or sugar products) will make my ***eating disorder symptoms…****						
ED diagnosis	578	2.60	8.30	71.63	12.80	4.67
A ban on "buy one get one free" deals for unhealthy foods and drinks (i.e., high fat, salt and/or sugar products) will make my ***other mental health symptoms…***						
ED diagnosis	515	4.47	9.13	76.12	6.41	3.88
No ED diagnosis	688	2.33	9.30	79.80	5.96	2.62

*This item was only administered to participants who have been diagnosed with an ED

n = number of participants; % = percentage; ED diagnosis= participants who have been diagnosed with an ED; No ED diagnosis= participants who have not been diagnosed with an ED

Table S11. Differences in acceptability and perceptions of banning "buy one get one free" deals for unhealthy food and drinks between participants who have and have not been diagnosed with an ED

Dependent variables	Disagree vs. neutral		Agree vs. neutral	
	**RRR**	**95% CI**	**RRR**	**95% CI**
"Buy one get one free" deals for unhealthy foods and drinks (i.e., high fat, salt and/or sugar products) should be banned (n = 915; ref = No ED diagnosis)				
ED diagnosis	1.01	0.69, 1.48	1.16	0.77, 1.73
A ban on "buy one get one free" deals for unhealthy foods and drinks (i.e., high fat, salt and/or sugar products) would be helpful to prevent from buying more unhealthy foods and drinks (n = 915; ref = No ED diagnosis)				
ED diagnosis	0.94	0.62, 1.43	0.87	0.59, 1.29
A ban on "buy one get one free" deals for unhealthy foods and drinks (i.e., high fat, salt and/or sugar products) will not provide benefits to people's health (n = 914; ref = No ED diagnosis)				
ED diagnosis	0.97	0.66, 1.42	1.19	0.80, 1.78
**Dependent variables**	**Worse vs. neutral**		**Better vs. neutral**	
	**RRR**	**95% CI**	**RRR**	**95% CI**
A ban on "buy one get one free" deals for unhealthy foods and drinks (i.e., high fat, salt and/or sugar products) will make my ***other mental health symptoms*** *(n = 873; ref = No ED diagnosis)*				
ED diagnosis	1.07	0.68, 1.68	1.56	0.98, 2.47

*p < .05; **p < .01; ***p < .001

RRR = relative risk ratio; CI = confidence interval; ref = reference group; ED diagnosis = participants who have been diagnosed with an ED; No ED diagnosis = participants who have not been diagnosed with an ED

Multinomial logistic regression models were developed for each item of acceptability and perceptions of the policy, adjusting for age, gender, ethnicity, education, tertiles of equivalised household income, and BMI category.

Table S12. Analyses examining differences in acceptability and perceptions of mandatory calorie labelling on menus between participants who have and have not been diagnosed with an ED using inverse probability weighting approach

Dependent variables	Disagree vs. neutral		Agree vs. neutral	
	**RRR**	**95% CI**	**RRR**	**95% CI**
Businesses like restaurants, fast food outlets and coffee shops should be required to display the calorie content of their foods on menus and menu boards (n = 915; ref = No ED diagnosis)				
ED diagnosis	2.06	1.28, 3.33**	1.14	0.73, 1.78
Calorie information on menus and menu boards will be helpful when deciding what I want to choose when eating out (n = 913; ref = No ED diagnosis)				
ED diagnosis	1.14	0.66, 1.99	0.77	0.46, 1.30
I will feel anxious if I see calorie information on menus and menu boards when eating out (n = 915; ref = No ED diagnosis)				
ED diagnosis	0.44	0.28, 0.69***	1.40	0.89, 2.20
*Compared to eating out without calorie labelling information, calorie labelling will make me feel **more guilty** when eating out (n = 915; ref = No ED diagnosis)*				
ED diagnosis	0.80	0.50, 1.28	1.72	1.11, 2.65*
*Compared to eating out without calorie labelling information, calorie labelling will make me feel **less guilty** when eating out (n = 915; ref = No ED diagnosis)*				
ED diagnosis	2.04	1.39, 2.99***	2.15	1.37, 3.37**
*Compared to eating out without calorie labelling, calorie labelling will make me feel **more afraid** about eating out (n = 915; ref = No ED diagnosis)*				
ED diagnosis	0.71	0.47, 1.07	1.98	1.28, 3.04**
*Compared to eating out without calorie labelling, calorie labelling will make me feel **less afraid** about eating out (n = 914; ref = No ED diagnosis)*				
ED diagnosis	1.63	1.16, 2.30**	1.70	1.13, 2.55*
If a menu without calorie information was also available, I would prefer to use that when eating out (n = 914; ref = No ED diagnosis)				
ED diagnosis	1.99	1.28, 3.10**	2.63	1.70, 4.06***
If a menu without calorie information was also available, I would feel comfortable asking for it when eating out (n = 915; ref = No ED diagnosis)				
ED diagnosis	1.49	0.99, 2.25	1.20	0.78, 1.86
**Dependent variables**	**Worse vs. neutral**		**Better vs. neutral**	
	**RRR**	**95% CI**	**RRR**	**95% CI**
*Seeing calorie information on menus or menu boards when eating out will make my **other mental health symptoms** (n = 872; ref = No ED diagnosis)*				
ED diagnosis	2.48	1.78, 3.47***	1.87	1.20, 2.94**

*p < .05; **p < .01; ***p < .001

RRR = relative risk ratio; CI = confidence interval; ref = reference group; ED diagnosis = participants who have been diagnosed with an ED; No ED diagnosis = participants who have not been diagnosed with an ED

Multinomial logistic regression models were developed for each item of acceptability and perceptions of the policy, adjusting for age, gender, ethnicity, education, tertiles of equivalised household income, and BMI category.

Table S13. Differences in acceptability and perceptions of mandatory calorie labelling on menus between participants who have and have not been diagnosed with an ED (models include additional adjustments for ED and mental health symptomology) using inverse probability weighting approach

Dependent variables	Disagree vs. neutral		Agree vs. neutral	
	RRR	95% CI	RRR	95% CI
Businesses like restaurants, fast food outlets and coffee shops should be required to display the calorie content of their foods on menus and menu boards (n = 903; ref = No ED diagnosis)				
ED diagnosis	1.93	1.17, 3.21*	0.98	0.61, 1.56
Calorie information on menus and menu boards will be helpful when deciding what I want to choose when eating out (n = 901; ref = No ED diagnosis)				
ED diagnosis	1.04	0.57, 1.90	0.61	0.35, 1.09
I will feel anxious if I see calorie information on menus and menu boards when eating out (n = 903; ref = No ED diagnosis)				
ED diagnosis	0.47	0.29, 0.75**	1.18	0.74, 1.89
Compared to eating out without calorie labelling information, calorie labelling will make me feel ***more guilty*** *when eating out (n = 903; ref = No ED diagnosis)*				
ED diagnosis	0.79	0.49, 1.29	1.44	0.91, 2.27
Compared to eating out without calorie labelling information, calorie labelling will make me feel ***less guilty*** *when eating out (n = 903; ref = No ED diagnosis)*				
ED diagnosis	1.87	1.26, 2.78**	1.78	1.11, 2.85*
Compared to eating out without calorie labelling, calorie labelling will make me feel ***more afraid*** *about eating out (n = 903; ref = No ED diagnosis)*				
ED diagnosis	0.76	0.49, 1.17	1.67	1.06, 2.63*
Compared to eating out without calorie labelling, calorie labelling will make me feel ***less afraid*** *about eating out (n = 902; ref = No ED diagnosis)*				
ED diagnosis	1.54	1.08, 2.21*	1.52	0.99, 2.35
If a menu without calorie information was also available, I would prefer to use that when eating out (n = 902 ref = No ED diagnosis)				
ED diagnosis	1.59	1.02, 2.50*	2.11	1.35, 3.30**
If a menu without calorie information was also available, I would feel comfortable asking for it when eating out (n = 903; ref = No ED diagnosis)				
ED diagnosis	1.21	0.80, 1.85	1.16	0.74, 1.82
**Dependent variables**	**Worse vs. neutral**		**Better vs. neutral**	
	**RRR**	**95% CI**	**RRR**	**95% CI**
Seeing calorie information on menus or menu boards when eating out will make my ***other mental health symptoms*** *(n = 860; ref = No ED diagnosis)*				
ED diagnosis	1.88	1.33, 2.66***	1.47	0.92, 2.34

*p < .05; **p < .01; ***p < .001

RRR = relative risk ratio; CI = confidence interval; ref = reference group; ED diagnosis = participants who have been diagnosed with an ED; No ED diagnosis= participants who have not been diagnosed with an ED

Multinomial logistic regression models were developed for each item of acceptability and perceptions of the policy, adjusting for age, gender, ethnicity, education, tertiles of equivalised household income, BMI category, ED symptoms from EDE-Q7 (shape/weight overvaluation, body dissatisfaction), and mental health symptoms from PHQ-4.

Additional findings when IPW approach was used to address missing observations for the analyses of the perceptions of calorie labelling policy:

- We found no statistically significant differences in the perceptions of calorie labelling policy based on the status of current ED diagnosis (current vs. past diagnosis) when IPW approach was used, similar to the findings presented in Table S5 *(full findings are not presented)*.
- We found no statistically significant associations between ED diagnoses (yes vs. no) and opinions on using and asking for a menu with calorie labelling when IPW approach was used, similar to the findings presented in Table S6 *(full findings are not presented)*.
- We also used IPW approach in examining the associations between ED diagnoses (yes vs. no) and perceived negative effects of calorie labelling on the current ED and mental health symptoms (worse vs. neutral) (see Table 4 in the main document for the comparison). Findings from using IPW approach indicated that the associations between the following ED diagnoses and perceived negative effects of calorie labelling on current ED symptoms were significant at $p < .05$: anorexia nervosa (RRR = 2.20; 95% CI = 1.04, 4.63; p = .039), bulimia nervosa (RRR = 2.42, 95% CI = 1.24, 4.71; p = .01) *(full findings are not presented)*.

Table S14. Differences in acceptability and perceptions of banning advertisements of unhealthy food and drinks online and before 9 pm on TV between participants who have and have not been diagnosed with an ED using inverse probability weighting approach

Dependent variables	Disagree vs. neutral		Agree vs. neutral	
	**RRR**	**95% CI**	**RRR**	**95% CI**
Marketing and advertising of unhealthy foods and drinks (i.e., high fat, salt and/or sugar products) should be banned online and before 9 pm on TV (n = 915; ref = No ED diagnosis)				
ED diagnosis	0.98	0.65, 1.48	0.86	0.60, 1.21
A ban on marketing and advertising of unhealthy foods and drinks (i.e., high fat, salt and/or sugar products) online and before 9 pm on TV will be helpful to prevent children from seeing more unhealthy food and drink advertisements (n = 915 ref = No ED diagnosis)				
ED diagnosis	0.87	0.52, 1.46	0.68	0.44, 1.06
A ban on marketing and advertising of unhealthy foods and drinks (i.e., high fat, salt and/or sugar products) online and before 9 pm on TV will not provide benefits to children's health (n = 914; ref = No ED diagnosis)				
ED diagnosis	0.69	0.48, 0.99*	0.81	0.54, 1.22
**Dependent variables**	**Worse vs. neutral**		**Better vs. neutral**	
	**RRR**	**95% CI**	**RRR**	**95% CI**
A ban on marketing and advertising of unhealthy foods and drinks (i.e., high fat, salt and/or sugar products) online and before 9 pm on TV will make my ***other mental health symptoms*** *(n = 873; ref = No ED diagnosis)*				
ED diagnosis	1.59	0.65, 3.92	1.26	0.81, 1.96

*p < .05; **p < .01; ***p < .001

RRR = relative risk ratio; CI = confidence interval; ref = reference group; ED diagnosis = participants who have been diagnosed with an ED; No ED diagnosis = participants who have not been diagnosed with an ED

Multinomial logistic regression models were developed for each item of acceptability and perceptions of the policy, adjusting for age, gender, ethnicity, education, tertiles of equivalised household income, and BMI category.

Table S15. Differences in acceptability and perceptions of banning "buy one get one free" deals for unhealthy food and drinks between participants who have and have not been diagnosed with an ED using inverse probability weighting approach

Dependent variables	Disagree vs. neutral		Agree vs. neutral	
	**RRR**	**95% CI**	**RRR**	**95% CI**
"Buy one get one free" deals for unhealthy foods and drinks (i.e., high fat, salt and/or sugar products) should be banned (n = 915; ref = No ED diagnosis)				
ED diagnosis	1.03	0.71, 1.51	1.18	0.79, 1.77
A ban on "buy one get one free" deals for unhealthy foods and drinks (i.e., high fat, salt and/or sugar products) would be helpful to prevent from buying more unhealthy foods and drinks (n = 915 ref = No ED diagnosis)				
ED diagnosis	0.93	0.61, 1.40	0.86	0.59, 1.27
A ban on "buy one get one free" deals for unhealthy foods and drinks (i.e., high fat, salt and/or sugar products) will not provide benefits to people's health (n = 914; ref = No ED diagnosis)				
ED diagnosis	0.97	0.66, 1.44	1.23	0.82, 1.83
**Dependent variables**	**Worse vs. neutral**		**Better vs. neutral**	
	**RRR**	**95% CI**	**RRR**	**95% CI**
A ban on "buy one get one free" deals for unhealthy foods and drinks (i.e., high fat, salt and/or sugar products) will make my ***other mental health symptoms*** *(n = 873; ref = No ED diagnosis)*				
ED diagnosis	1.07	0.69, 1.68	1.58	1.00, 2.52

*p < .05; **p < .01; ***p < .001

RRR = relative risk ratio; CI = confidence interval; ref = reference group; ED diagnosis = participants who have been diagnosed with an ED; No ED diagnosis = participants who have not been diagnosed with an ED

Multinomial logistic regression models were developed for each item of acceptability and perceptions of the policy, adjusting for age, gender, ethnicity, education, tertiles of equivalised household income, and BMI category.
